# Scientific breeding of winter bread wheat in the Non-Сhernozem zone of Russia: the history, methods and results

**DOI:** 10.18699/VJ21.53-o

**Published:** 2021-07

**Authors:** B.I. Sandukhadze, R.Z. Mamedov, M.S. Krakhmalyova, V.V. Bugrova

**Affiliations:** Federal Research Center “Nemchinovka”, Novoivanovskoye, Odintsovo, Moscow Region, Russia; Federal Research Center “Nemchinovka”, Novoivanovskoye, Odintsovo, Moscow Region, Russia; Federal Research Center “Nemchinovka”, Novoivanovskoye, Odintsovo, Moscow Region, Russia; Federal Research Center “Nemchinovka”, Novoivanovskoye, Odintsovo, Moscow Region, Russia

**Keywords:** winter bread wheat, breeding, variety, winter hardiness, yield, lodging resistance, short stemmed plants, озимая мягкая пшеница, селекция, сорт, зимостойкость, урожайность, устойчивость к полеганию, короткостебельность

## Abstract

The article describes the main stages and achievements of the breeding of winter bread wheat (Triticum aestivum L.) in the Non-Chernozem zone for more than a century. The beginning of breeding work was laid by D.L. Rudzinsky on the experimental field of the Moscow Agricultural Institute. Beginning from the 1940s, under the leadership of Academician N.V. Tsitsin, and then Prof. G.D. Lapchenko, the method of distinct hybridization with blue wheatgrass (Agropyron glaucum (Desf. ex DC.) Roem. & Schult.) was actively used. The resulting wheat-wheatgrass hybrids had an average winter hardiness, increased grain quality and productivity. Cultivar Zarya developed in the 1970s (by individual selection from the F3 cross combination of cv. Mironovskaya 808×line 126/65 (in the pedigree of this line, there is a wheat-wheatgrass hybrid PPG 599)) had a high yield and was widely used in further crosses. In the 1980s, Academician B.I. Sandukhadze achieved a significant increase in yield by using the method of intermittent backcrosses due to the producing of varieties with a new morphoecotype (cvs Inna, Pamyati Fedina, etc.), namely, winter-hardy, short stemmed (dwarf), and productive. Cultivar Moskovskaya 39 (registration in 1999) was referred to strong wheat, with a stable protein content of 15–16 %, gluten 30–35 %. Produced in the 2000s, cvs Moskovskaya 56, Nemchinovskaya 57, Galina, Nemchinovskaya 24, Nemchinovskaya 17, and Moskovskaya 40 have a high adaptability to the environment of the region; give a high yield and quality of grain. The area of crops of these cultivars in Russia occupies more than 2 million ha. The current trends in wheat breeding are indicated, the production yield of commercial cultivars of breeding by the Federal Research Center “Nemchinovka” over 12.0 tons per ha and the protein content in the grain up to 17 % are shown. As a result of succession, originality and application of the methodology of scientific breeding, the yield of winter bread wheat in the period from the beginning of the last century to the present has increased from 1.0 to 12.0 and more tons per ha.

## Abstract (ru)

Рассмотрены основные этапы и достижения селекции озимой мягкой пшеницы (Triticum aestivum L.) в Нечерноземной зоне более чем за столетний период. Начало селекционной работы в регионе было положено Д.Л. Рудзинским на опытном поле Московского сельскохозяйственного института. С 1940-х гг. под руководством академика Н.В. Цицина, а затем профессора Г.Д. Лапченко активно применялся метод отдаленной гибридизации пшеницы с пыреем сизым (Agropyron glaucum (Desf. ex DC.) Roem. & Schult.). Полученные пшенично-пырейные гибриды (ППГ) обладали средней зимостойкостью, повышенным качеством зерна и продуктивностью. Созданный в 1970-х гг. сорт Заря (индивидуальным отбором из F3 гибридной комбинации сорт Мироновская 808×линия 126/65, в родословной этой линии присутствует ППГ 599) имел высокую урожайность и широко использовался в дальнейших скрещиваниях. В 1980-е гг. академик Б.И. Сандухадзе методом прерывающихся беккроссов добился значительного увеличения урожайности за счет выведения сортов нового морфоэкотипа (Инна, Памяти Федина и др.) – зимостойких, короткостебельных, продуктивных. Сорт Московская 39 (районирован в 1999 г.) относится к сильным пшеницам со стабильным содержанием белка (15–16 %) и клейковины (30–35 %). Созданные в 2000-х гг. сорта Московская 56, Немчиновская 57, Галина, Немчиновская 24, Немчиновская 17, Московская 40 характеризуются высокой адаптивностью к условиям региона, высокой урожайностью и качеством зерна. Площади посевов этих сортов в России занимают более 2 млн га. Обозначены актуальные направления селекции озимой мягкой пшеницы, показана производственная урожайность сортов селекции ФГБНУ «ФИЦ «Немчиновка» свыше 12.0 т/га и содержание белка в зерне до 17 %. Благодаря преемственности, оригинальности и методологии научной селекции, урожайность озимой пшеницы в регионе с начала прошлого века до настоящего времени выросла с 1.0 до 12.0 т/га и более.

## Introduction

At the beginning of the last century, wheat (Triticum L.) was not widely distributed in the Non-Chernozem zone of Russia. In production, “brown” breads were grown: winter rye and oats. Local varieties were cultivated from wheat and, as a rule, economic characteristics instead of names – “local”, “winter”, “spring”. These cultivars of folk breeding were populations consisting of a mixture of cultivars, and sometimes species (Flaksberger, 1929).

The promotion of wheat culture took place in the first years of the XX century and was associated with the activities of the Committee for Plant Acclimatization at the Moscow Society of Agriculture. In the Non-Chernozem zone of Russia, scientific breeding of wheat and a number of other crops was started at the Shatilov Experimental Station (organized in 1896). In 1903, the foundations of scientific breeding of field crops were laid at the experimental field of the Moscow Agricultural Institute (now the Russian State Agrarian University – Moscow Timiryazev Agricultural Academy), where D.L. Rudzinsky, S.I. Zhegalov, A.G. Lorkh, N.I. Vavilov and other outstanding scientists worked (Goncharov, 2005; Elina, 2007). More than 3,000 winter wheat variety samples from the Russian Empire, Europe, and North America were studied at the experimental field of the Moscow Agricultural Institute (Flaksberger, 1929). The signs, for the manifestation of which it was advisable to conduct the selection of elite plants, were determined. Assessing the twenty-year work of the Moscow Breeding Station of the Moscow Agricultural Academy, N.I. Vavilov (1929) noted the volume of the analyzed material. At the same time, he pointed to the fact that the conducted selections did not provide significant changes in the expression of traits and properties in the cultivars relative to the original selected populations, which, in his opinion, indicated the need to use interspecific and intergenerational hybridization more widely

In the 1930s, the creation of winter-hardy, resistant to damping off and soaking plastic cultivars, immune to powdery mildew, brown rust and fusarium was continued. In 1940, Academician N.V. Tsitsin organized a laboratory of wheatwheatgrass hybrids at the Zonal Institute of Grain Farming in the Non-Chernozem Zone (later NIISH CRNZ, Moscow NIISH “Nemchinovka”, now the Federal Research Center “Nemchinovka”) and continued the work on remote hybridization of wheat with wild wheatgrass (Agropyron glaucum (Desf. ex DC.) Roem. & Schult. = syn. Thinopyrum intermedium (Host) Barkworth & D.R. Dewey) for the production of winter bread wheat cultivars (Lapchenko, 1967). From 42-chromosomal forms of wheat-wheatgrass hybrids (PPG) with the wheat ear type, N.V. Tsitsin and G.D. Lapchenko first derived winter cultivars of bread wheat based on PPG 599 and PPG 186. The plants showed an average level of winter hardiness, individual breeding numbers contained up to 19 % protein in the grain. These cultivars were zoned in 18 regions and republics of the Non-Chernozem zone of Russia.

In the laboratory of winter bread wheat breeding organized by E.T. Varenitsa in 1951, intraspecific multi-stage hybridization of remote ecological and geographical forms with the use of selective fertilization was widely used in the Research Institute of Agricultural Research and Development of CRNZ. The best cultivars zoned in the area were used as maternal forms, and the cultivars with high yield, winter hardiness, resistance to pathogens and lodging, taken from other ecological and geographical zones, were used as paternal forms (Varenitsa, 1971).

The positive results of the breeding of winter bread wheat in the 1970s are associated with the creation of the cv. Zarya, obtained by individual selection from F3 hybrids of the combination of crossing the Ukrainian cultivar Mironovskaya 808 with the line 126/65 (in the pedigree of which there is a PPG 599). In 1978, the cv. Zarya was zoned. The maximum area of its cultivation was 530 thousand hectares (Varenitsa, 1987). Later, by the individual selection of the cv. Zarya the cv. Yantarnaya 50 (zoned in 1985), characterized by high productivity, large grain, high weight of 1000 grains, but with weak winter hardiness was obtained.

## Breeding of intensive type cultivars

In 1984, B.I. Sandukhadze headed the breeding of winter bread wheat in the NIISH CRNZ. In place of the cv. Mironovskaya 808, which was widely cultivated in the Non-Chernozem zone, it was necessary to create more technologically advanced intensive cultivars with high grain quality, more resistant to lodging, unfavorable overwintering conditions, and fungal diseases. It was necessary to overcome the negative relationship between high yield and high winter hardiness, as well as high winter hardiness and short-stemmed vegetation. The best short-stem donor was recognized as the Krasnodar Dwarf 1, bred in the Krasnodar Research Institute of Agricultural Sciences. Hybrids from crossing the cv. Mironovskaya 808 with it consistently inherited low plant height and increased winter hardiness over the years.

To obtain short-stemmed and winter-hardy varieties of intensive type, the method of intermittent backcrosses was used, which became the basis for cultivars of a new morphoecotype (Sandukhadze et al., 1996). The next backcross involved plants selected from families of F3 hybrids for optimal overwintering, plant height and productivity. After three backcrosses, the breeding samples were compared for winter hardiness with the recurrent parent. Selection was more effective in BC3–BC4 populations with better productivity. In theory, this method of breeding allowed us to count on a higher probability of obtaining new combinations of genes in the offspring of the next backcross, in practice – on a higher efficiency of the entire selection process. The height of the plants increased depending on the number of backcrosses in F1 hybrids. By this method, seven cultivars were obtained in Nemchinovka: Nemchinovskaya 52, Nemchinovskaya 86, Moskovskaya nizkostebelnaya, Moskovskaya 70, Inna, Pamyati Fedina and Nemchinovskaya 25, zoned in 12 regions and republics of the RSFSR. The cvs. Inna and Pamyati Fedina outperformed the others by 1.0 t per ha in productivity (Sandukhadze et al., 2001). Created by the early 1990s, a series of cultivars of a new morphoecotype with a high productivity potential and a yield exceeding 1.0 t per ha or more relative to the long-stemmed standard cultivar, adapted to the conditions of the central regions of the Non-Chernozem region, became a breakthrough in the breeding of winter bread wheat for this region.

As a result of purposeful breeding work, a number of cultivars of winter bread wheat were created in the FRC “Nemchinovka”, currently occupying a total of more than 2 million hectares (Table 1). Since the end of the 1990s, the cvs. Moskovskaya 39 (1999), Nemchinovskaya 24 (2006), Moskovskaya 56 (2008), Nemchinovskaya 57 (2009), Moskovskaya 40 (2011), Nemchinovskaya 17 (2013), Nemchinovskaya 85 (2021) and Moskovskaya 82 (created together with the Nizhny Novgorod NIISH, a branch of the N.V. Rudnitsky Federal Agricultural Research Center of the North-East, zoned in 2021) have been zoned in more than 35 regions of the Russian Federation. The cv. Moskovskaya 39 was obtained by selection from the hybrid combination Obriy×Yantarnaya 50 and has the property of direct translocation of nitrogen-containing compounds from the soil to the grain during its filling, which enhances the biosynthesis of spare proteins. The uniqueness of the cultivar is that, in all quality indicators, it consistently exceeds all previously zoned cultivars, and the protein content in the grain is higher. Thanks to the cv. Moskovskaya 39, stable production of grain for baking in densely populated Central Russia has become possible (Sandukhadze et al., 2016). The areas of crops of the listed cultivars were indirectly calculated according to the data of the Russian Agricultural Center for 2017–2019 by the number of sown seeds based on the seeding rate of 200 kg/ha. The actual acreage is much larger.

**Table 1.**
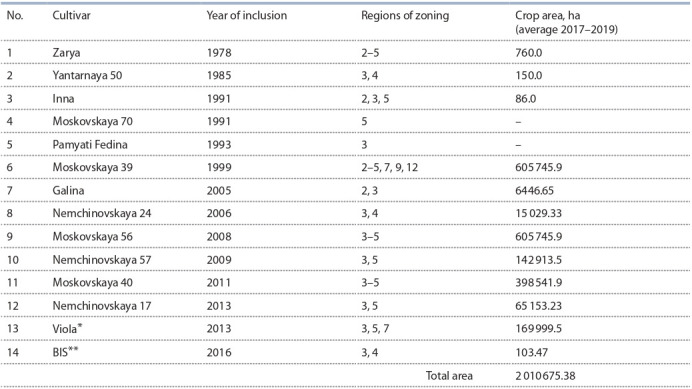
Cultivars of breeding of FRC “Nemchinovka” (Laboratory of breeding and primary seed production of winter wheat) included in the “State Register of breeding achievements approved for use” in 2020 Jointly with the Federal Scientific Agroengineering Center VIM; ** jointly with the Verkhnevolzhsky FARC.

Currently, the State competitive cultivar testing for the cv. Moskovskaya 27, which has been transferred in 2019, is taking place.

**Fig. 1.**
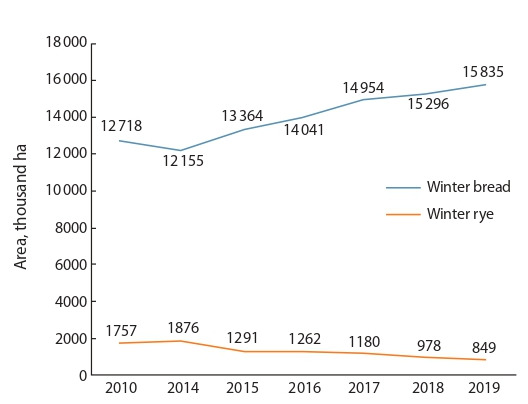
Sown areas of winter bread wheat and winter rye in farms of all categories of the Russian Federation.

According to the 1916 census, the areas under winter crops in the provinces of the center of the Non-Chernozem region were as follows: winter rye – 1,196,448 ha (99.7 %), winter wheat – 3,120 (0.3 %) (Sekun, 1954). Now the situation is exactly the opposite. According to Rosstat (rosstat.gov.ru), the share of winter bread wheat in the grain crop wedge is constantly increasing (Fig. 1).

By 2050, global demand for agricultural crops is projected to roughly double, driven by population growth, meat and dairy consumption, and the use of biofuels (Godfray et al., 2010; Tilman et al., 2011). Wheat is one of the main food crops around the world, and the need for new varieties of winter bread wheat is particularly relevant today (Sandukhadze, 2010; Ray et al., 2012; Kudryashov et al., 2016). The Laboratory of breeding and primary seed production of winter wheat at the FRC “Nemchinovka” has a priority role in its breeding for the Non-Chernozem zone and other regions of the Russian Federation. Next, we will consider the main directions of winter wheat breeding. In addition to these areas, work on early maturity, resistance to diseases and pests, drought resistance and other signs and properties is underway.

## Breeding for frost and winter hardiness

Many authors note that breeding for adaptability allows combining high productivity and resistance to limiting environmental factors in the genotype of the cultivar (Romanenko, Lavrenchuk, 2011). In natural conditions, selection on this basis is possible only in severe winters, with early thaws in the spring, return frosts and other unfavorable factors.

In the FRC “Nemchinovka”, to maintain a high level of winter hardiness, a local zoned variety is necessarily used as one of the parents in pair and backcross crosses. The cvs. Mironovskaya 808, Pamyati Fedina, Moskovskaya 56 and Nemchinovskaya 57 serve as donors of frost and winter hardiness for the Non-Chernozem zone.

## Breeding for short-stemmed plants

Resistance to lodging is one of the priority areas for improving modern cultivars. Successful hybridization and subsequent breeding can only be based on attentive attitude to the forms of local origin, along with a constant search for sources and donors of useful traits and properties in the global gene pool (Likhenko, 2008). Russian breeders pay attention to the search, identification and creation of new highly productive and short-stemmed source material for winter wheat and other grain crops (Samofalova, 2016; Medvedev et al., 2017; Dyachuk et al., 2018).

Numerous studies have found that the lodging of crops not only reduces the yield, but also negatively affects the baking and sowing qualities of grain (Packa et al., 2015; Khobra et al., 2019; Ageeva et al., 2020). The main method of increasing resistance to lodging is to reduce the height of plants. The donor of this trait for winter cultivars of the Non-Chernozem zone, as a rule, is a geographically distant form. The breeding advantage of short-stemmed forms can be attributed to their high productive bushiness, the disadvantages – low winter hardiness and weight of 1000 grains (Sandukhadze et al., 1996). In the late 1980s, the Krasnodar Dwarf 1, a mutant obtained from the cv. Bezostaya 1 under the influence of nitrosomethyl urea, which is a donor of the Rht-11 gene, was used in crosses (Divashuk et al., 2012). This mutant is present in the pedigree of the cvs. Inna and Pamyati Fedina, which, in turn, were one of the parent forms of the cvs. Nemchinovskaya 24, Moskovskaya 56, Nemchinovskaya 17, Galina, Nemchinovskaya 57, Nemchinovskaya 85 and Moskovskaya 27

**Fig. 2.**
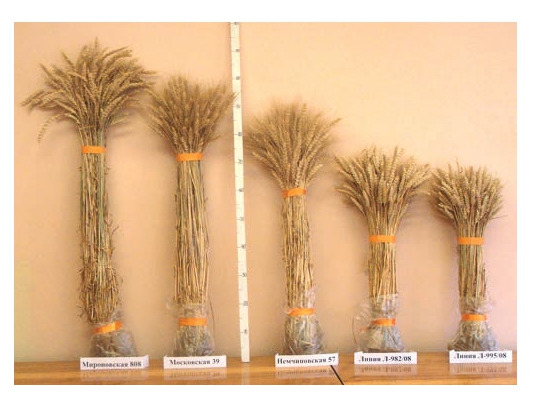
Decrease in plant height of winter wheat cultivars as a result of breeding.

Since 2008, a sample from Italy called Agapik has played an important role in breeding for lodging resistance. The height of the plants is 65–70 cm. Thousands of lines were worked out in crosses with it. The cvs. Nemchinovskaya 85 (Agapik× Pamyati Fedina) and Moskovskaya 27 (Lutescens 982/08× Moskovskaya 56) have this sample in their pedigree. The Lutescens 982/08 line is a paired Agapik×Pamyati Fedina hybrid (Fig. 2).

## Breeding for grain quality

Recently, producers have been interested not only in high yields, but also in different cultivars, including those that can meet the market needs for increasing the protein content and dough weight (Vitale et al., 2020). The problem of wheat grain quality is an integrating indicator of the interaction of the variety genotype, natural and ecological features, agrotechni- cal and organizational and economic conditions of cultivation (Rozbicki et al., 2014)

The attribute “total protein content in grain” is controlled polygenically (Mitrofanova, Khakimova, 2016). At present, wheat has many major and minor loci that affect the amount of protein in the grain, the prominence of which is not stable in its manifestation. The protein content in the grain and the yield are negatively correlated, which complicates the breeding to increase the prominence of both traits at the same time.

A distinctive feature of the winter bread wheat cultivars of the Nemchinovsky breeding is their high quality indicators. To create cultivars with such a level of protein and gluten content, we use the cvs. Moskovskaya 39 and Moskovskaya 40 (obtained by individual selection from Moskovskaya 39) in complex hybrid combinations, paired crosses, and in individual selections for the possibility of combining quality indicators with high productivity and adaptability of the new breeding material in one genotype (Sandukhadze et al., 2006).

## Breeding for yield

The ultimate goal of wheat production is to produce high grain yields. Yield is a polygenic trait, and its formation is influenced by many factors. The main components of the crop: the number of productive stems per 1 m2 and the weight of grain per ear (number of grains, weight of 1000 grains) (Krasnova, Zhivoderova, 2003; Goncharov, 2012; Voronchikhin et al., 2018). It should be noted that modern breeding methods, such as genotyping, selection using molecular markers, genome editing, and others, are ineffective without field testing of the created material (Hickey et al., 2019; Lozada et al., 2020). To obtain new cultivars, various types of crossing are carried out (simple, complex, backcrosses), the parent forms are selected both according to the ecological and geographical principle, and according to the elements of the crop structure. Modern cultivars of the Center’s breeding are high-yielding, adapted to the conditions of the region, and actively used in crosses for these characteristics. The level of productivity of the Nemchinovsky breeding cultivars obtained in field tests is presented in Table 2.

**Table 2.**
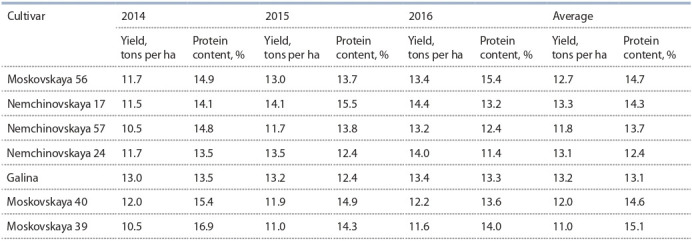
Yield and protein content of winter bread wheat cultivars under high-intensity cultivation technology (2014–2016)

**Fig. 3.**
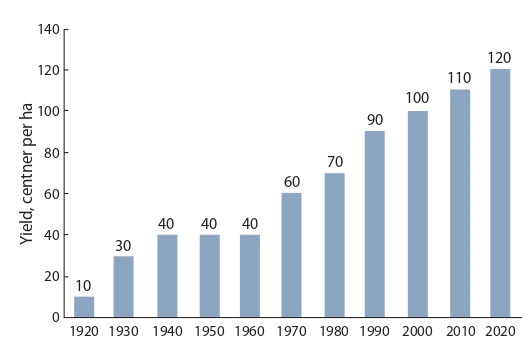
. Results of breeding for the yield of winter bread wheat in the NonChernozem zone for the centennial period (1920–2020).

Figure 3 shows the average yield of cultivated winter wheat cultivars in the Non-Chernozem zone. Scientific breeding of winter wheat allowed to increase the productivity of cultivated cultivars by more than 10 times (see Fig. 3). Since the 1970s, the main cultivated cultivars in the Non-Chernozem zone have been cultivars of our institute’s breeding

## Conclusion

Leading domestic breeders developed and effectively applied advanced for their time methods and schemes of the breeding process, such as the hybridization of cultivars with identified economically valuable traits and properties, the remote hybridization of bread wheat with wheatgrass and PPG to obtain winter-hardy, disease-resistant plants with increased grain quality, the crossing of geographically distant forms, the use of intermittent backcrosses and the creation of a new morphoecotype of the cultivar (short-stemmed, resistant to lodging, with increased winter hardiness and grain quality), which allowed to provide Nechernozemye, a densely populated region of the Russian Federation, with its own grain. New cultivars of winter wheat of the Federal Research Center “Nemchinovka” have a high adaptability to the conditions of the region. This allows us to produce consistently high grain yields with good baking qualities. Over a hundred years of scientific breeding, the yield of soft winter wheat cultivars has increased to 14.0 tons per ha and is almost 10 times higher than the yield of cultivars of the first stages of breeding in the region.

## Conflict of interest

The authors declare no conflict of interest.

## References

Ageeva E.V., Leonova I.N., Likhenko I.E. Lodging in wheat: genetic
and environmental factors and ways of overcoming. Vavilovskii
Zhurnal Genetiki i Selektsii = Vavilov Journal of Genetics and
Breeding. 2020;24(4):356-362. DOI 10.18699/VJ20.628. (in Russian)

Divashuk M.G., Vasiliev A.V., Bespalova L.A., Karlov G.I. Identity of
the Rht-11 and Rht-B1e reduced plant height genes. Russ. J. Genet.
2012;48(7):761-763. DOI 10.1134/S1022795412050055.

Dyachuk T.I., Kibkalo I.A., Pominov A.V., Khomyakova O.V., Akinina V.N. The promising lines in the breeding work with Triticale for
the Povolzhie conditions. Zernovoe Hozâjstvo Rossii = Grain Economy of Russia. 2018;5:39-43. DOI 2079-8725-2018-59-5-39-43.
(in Russian)

Elina O.Yu. “Our teacher” Dionysy Leopoldovich Rudzinsky: Back
to the sources of plant breeding in Russia. Informatsionnyy Vestnik
VOGiS = The Herald of Vavilov Society for Geneticists and Breeding
Scientists. 2007;11(3/4):575-590. (in Russian)

Flaksberger K.A. Remarks on work with winter wheat. In: The Death
of Winter Bread and Measures to Prevent it. (Appendix No. 34 to
“Proceed. on Applied Botany, Genetics, and Breeding”). Leningrad:
VIPBiNK Publ., 1929;315-318. (in Russian)

Godfray H.Ch.J., Beddington J.R., Crute I.R., Haddad L., Lawrence D.,
Muir J.F., Pretty J., Robinson S., Thomas S.M., Toulmin C. Food
security: the challenge of feeding 9 billion people. Science. 2010;
327:812-818. DOI 10.1126/science.1185383.

Goncharov N.P. To the 250th anniversary of plant breeding in Russia.
Informatsionnyy Vestnik VOGiS = The Herald of Vavilov Society for
Geneticists and Breeding Scientists. 2005;9(3):279-289. (in Russian)

Goncharov N.P. Comparative Genetics of Wheat and its Relatives.
2nd ed. Novosibirsk: Acad. Publ. House “Geo”, 2012. (in Russian)

Hickey L.T., Haffer A., Robinson H., Jackson S.A., Leal-Bertioli S.C.M., Tester M., Gao C., Godwin I.D., Hayes B.J., Wulff B.B.H.
Breeding crops to feed 10 billion. Nat. Biotechnol. 2019;37(7):744-
754. DOI 10.1038/s41587-019-0152-9.

Khobra R., Sareen S., Meena B.K., Kumar A., Tiwari V.K., Singh G.P.
Exploring the traits for lodging tolerance in wheat genotypes.
Physiol. Mol. Biol. Plants. 2019;25(3):589-600. DOI 10.1007/s1229
8018-0629-x.

Krasnova L.I., Zhivoderova S.P. Formation of a productive stand in
zoned and promising winter wheat varieties of the Southern Ural region. In: Science and Bread: Theoretical and Practical Issues. 2003;
10:84-103. (in Russian)

Kudryashov I.G., Bespalova L.A., Ponomarev D.A. Relevance of varietal structures in winter wheat production in present conditions.
Zernovoe Hozâjstvo Rossii = Grain Economy of Russia. 2016;1:
9-13. (in Russian)

Lapchenko G.D. Application of the remote hybridization method in
winter wheat breeding. Selektsiya i Semenovodstvo = Breeding and
Seed Production. 1967;2:33-38. (in Russian)

Likhenko I.E. The use of the world gene pool and local varieties in
spring soft wheat breeding. Sibirskiy Vestnik Selskokhozyaystvennoy
Nauki = Siberian Herald of Agricultural Sciences. 2008;1:25-30. (in
Russian)

Lozada D.N., Carter A.H., Ward B.P. Gains through for grain yield in
a winter wheat breeding program. PLoS One. 2020;15(4):e0221603.
DOI 10.1371/journal.pone.0221603.

Medvedev A.M., Poma N.G., Osipov V.V., Liseenko E.N., Dyachenko E.V., Tupatilova O.V. The search of the sources of short stem to
grow tolerant to lodging varieties of winter Triticale for the Central
Non-Chernozem region. Zernovoe Hozâjstvo Rossii = Grain Economy of Russia. 2017;3:43-46. (in Russian)

Mitrofanova O.P., Khakimova A.G. New genetic resources in wheat
breeding for an increased grain protein content. Vavilovskii Zhurnal Genetiki i Selektsii = Vavilov Journal of Genetics and Breeding.
2016;20(4):545-554. DOI 10.18699/VJ16.177. (in Russian)

Packa D., Wiwart M., Suchowilska E., Dienkowska T. Morpho-anatomical traits of two lowest internodes related to lodging resistance
in selected genotypes of Triticum. Int. Agrophys. 2015;29:475-483.
DOI 10.1515/intag-2015-0053.

Ray D.K., Mueller N.D., West P.C., Foley J.A., Ramancuty N. Recent
patterns of crop yield growth and stagnation. Nat. Commun. 2012;
31:1293. DOI 1038/ncomms2296.

Romanenko A.A., Lavrenchuk N.F. Breeding of grain crops for resistance to abiotic stressors. Vestnik RASKhN = Herald of the Russian
Academy of Agricultural Sciences. 2011;1:17-21. (in Russian)

Rozbicki J., Ceglińska А., Gozdowski D., Wijata М. Influence of the
cultivar, environment and management on the grain yield and breadmaking quality in winter wheat. J. Cereal Sci. 2014;61:126-132.
DOI 10.1016/j.jcs.2014.11.001.

Samofalova N.E. Dreams and reality of the academician I.G. Kalinenko
in creation of durum winter wheat. Zernovoe Hozâjstvo Rossii =
Grain Economy of Russia. 2016;1:1-9. (in Russian)

Sandukhadze B.I. Winter wheat breeding as the most important factor
in increasing productivity and quality. Dostizheniya Nauki i Tekhniki
APK = Achievements of Science and Technology of AIC. 2010;11:
4-7. (in Russian)

Sandukhadze B.I., Kochetygov G.V., Bugrova V.V. Targeted breeding
of intensive winter wheat for Non-Chernozem conditions based on
the method of intermittent backcrosses. Selskokhozyaystvennaya
Biologiya = Agricultural Biology. 1996;1:13-26. (in Russian)

Sandukhadze B.I., Kochetygov G.V., Bugrova V.V. Winter wheat varieties Inna, Pamyati Fedina and Moskovskaya 39. In: The Main
Results of Scientific Research on Agriculture in the Central Region
of the Non-Chernozem Zone of Russia (70th Anniversary of the
Research Institute of Agriculture for the Central Non-Chernozem
Zone). Moscow: Nemchinovka, 2001;210-214. (in Russian)

Sandukhadze B.I., Kochetygov G.V., Bugrova V.V., Rybakova M.I.,
Berkutova N.S., Davydova E.I. Methodological bases of winter
wheat breeding for grain yield and quality in the central Non-Chernozem Zone of Russia. Selskokhozyaystvennaya Biologiya = Agricultural Biology. 2006;3:3-12. (in Russian)

Sandukhadze B.I., Rybakova M.I., Kochetygov G.V., Mamedov R.Z.,
Bugrova V.V., Sandukhadze K.E. Productivity and quality of winter
wheat varieties bred by Moscow NIISKH “Nemchinovka” and cultivated in central Russia. In: Zhuchenko Reading II. Food Security
of Agriculture in Russia in the XXI Century. Collection of scientific
works. Moscow, 2016;11(59):21-26. (in Russian)

Sekun P.F. Winter Wheat in the Non-Chernozem Zone. Moscow: Selkhozizdat Publ., 1954. (in Russian)
Tilman D., Balzer C., Hill J., Befort B.L. Global food demand and the
sustainable intensification of agriculture. Proc. Natl. Acad. Sci. USA.
2011;108:20260-20264. DOI 10.1073/pnas.1116437108.

Varenitsa E.T. Methods, results, and prospects of work on raising
highly productive varieties of winter wheat for the Non-Chernozem Zone. In: Breeding and Varietal Agrotechnology of Winter Wheat. (Proceedings of VASKHNIL). Moscow, 1971;194-205. (in
Russian)

Varenitsa E.T. Winter wheat. In: Nettevich E.D. (Ed.). Highly Productive Varieties of Grain Crops for the Non-Chernozem Zone. Moscow: Moskovskii Rabochii Publ., 1987;5-43. (in Russian)

Vavilov N.I. Botanical and geographical considerations on the possibility of promoting winter wheat culture in the USSR. In: The Death
of Winter Crops and Measures to Prevent it. (Appendix No. 34 to
“Proceed. on Applied Botany, Genetics and Breeding”). Leningrad,
1929;265-274. (in Russian)

Vitale J., Adam B., Vitale P. Economics of wheat breeding strategies:
focusing on Oklahoma hard red winter wheat. Agronomy. 2020;
10(2):238. DOI 10.3390/agronomy10020238.

Voronchikhin V.V., Pylnev V.V., Rubets V.S., Voronchikhina I.N. Yield
and elements of its structure of the winter hexaploid triticale collection in the Central region of the Non-Chernozem zone. Izvestiya Timiryazevskoi Selskokhozyaistvennoi Academii = Izvestiya of
Timiryazev Agricultural Academy. 2018;1:69-81. DOI 10.26897/
0021-342X-2018-1-69-81. (in Russian)

